# The lost art of preventive oncology

**DOI:** 10.3332/ecancer.2021.ed113

**Published:** 2021-08-17

**Authors:** Abeer Arain

**Affiliations:** Houston Methodist Hospital, 6565 Fannin street, Houston, TX 77030

**Keywords:** cancer, preventive medicine, oncologist, patient education

## Abstract

As the second most common cause of mortality worldwide, cancer is now prevalent in our society. The overall number of cases continue to rise each year and given the advancements in treatment strategies we now have a large population of cancer survivors. In the busy oncology clinic, patients often bring up the question of what caused their cancer. Oftentimes the answer is not very clear. This article is highlighting the role of oncology clinic in improving the awareness of overall health in patients and their families. The oncologist can play a crucial role in reducing the risk of malignancies in the family members of their patients. There is a need to have a quick discussion on the role of diet, exercise, vaccination against HPV, avoidance of smoking, alcohol, and other toxins such as pesticides in the oncology clinic.

Cancer is the leading cause of morbidity and mortality worldwide [1]. About 19.3 million new cases and almost 10.0 million cancer deaths occurred in 2020. Interestingly, only 5-10% of all cancers are caused by genetic mutations and up to 90% of cancers have their roots in the lifestyle and environmental factors [2, 3]. Nowadays, we see the diagnosis of cancer in the younger age group [4]. Colon cancer, once a disease of the elderly, is frequently being diagnosed in the 30's and 40's [4]. From the management standpoint, the traditional treatment of cancer includes surgery, chemotherapy and radiation therapy [5]. In today's era, we are broadening our horizons by bringing immunotherapy and targeted treatment strategies in our league [5]. However, in the midst of the advanced treatment modalities, the prevention of this deadly disease seems to lag behind [6].

Some of the common scenarios in the oncology clinic include: a patient with newly diagnosed cervical cancer coming for the follow-up with her daughter who is not vaccinated against HPV; a middle-aged man with colon cancer is in the clinic with his family members who are overweight; a patient with lung cancer is accompanied by his wife and there is a strong smell of cigarette smoke in the room. The health condition of our patient's family members is often ignored, whilst it can be a helpful clue in speculating the current or past environment at our patient's home.

In the usual oncology clinic, once the diagnosis of cancer is revealed, the oncologist, being an important driver of the cancer care, discusses the management plan in detail. A newly diagnosed case requires discussion on the introduction to the type of cancer, its epidemiology, treatment options, chemotherapy cycles, and the side-effects [7]. Over the course of their treatment, patients develop a rapport with their oncologist and often see them more than their primary care provider.

But how often does an oncologist talk about the etiology of the cancer? When the patients ask about the cause of their cancer, the usual answer given to them is that ‘we don’t really know for sure, sometimes it’s just bad luck’. In recent years, oncologists have made some progress in talking about smoking cessation to patients with lung or bladder cancer [8]. Patients with liver cancer are asked to cut down on the use of alcohol [8]. But is this enough? According to the literature, time constraints and limited clinic hours are common reasons for not bringing up a detailed discussion by the healthcare providers on lifestyle modifications [8]. Oncologists incline towards the patients’ primary care provider to discuss preventive medicine. On the other hand, primary care doctors have limited time to discuss the dietary modifications/exercise in their busy practice [8]. Studies have shown that there is better compliance and effect when the counseling comes from the provider, more so from the patient’s oncologist [8].

The cancer clinic can not only be a place for the management of cancers, but also an area where the prevention of cancer is highlighted. For a woman with cervical cancer coming to the clinic with her teenage daughter who is not vaccinated against HPV, it will take a few minutes for an oncologist to emphasize the important role of the HPV vaccine in preventing cervical cancers. Obesity is a risk factor for endometrial and colon cancer and an oncologist can play a vital role in highlighting the importance of weight loss and dietary modification so that the family members are aware of the steps that might help reduce the risk of developing malignancies.

## Conclusion

Our textbooks and journal articles begin with the discussion on cancer etiology, and as the sole provider of cancer care, oncologists can make a difference in the overall health of society by taking a few minutes to discuss the risk factors of cancer (such as obesity, unhealthy diet, HPV vaccination) with the patients and their family members who accompany them to the clinic follow-ups.

## Recommendation

Preventive oncology is more important than ever in the current era of processed food and sedentary lifestyles. The role of an oncology department is important in highlighting the preventive causes of cancer. Health promotion, patient education and preventive medicine must not be limited to the primary care providers’ office.

## Disclosures

The author has no disclosures or disclaimers related to the manuscript.

## Conflicts of interest

The author has no conflicts of interest.

## Funding statement

There was no funding support for this manuscript.
